# Molecular diagnosis of non-syndromic hearing loss patients using a stepwise approach

**DOI:** 10.1038/s41598-021-83493-6

**Published:** 2021-02-17

**Authors:** Jing Wang, Jiale Xiang, Lisha Chen, Hongyu Luo, Xiuhua Xu, Nan Li, Chunming Cui, Jingjing Xu, Nana Song, Jiguang Peng, Zhiyu Peng

**Affiliations:** 1 Department of Obstetrics and Gynecology, The First Affiliated Hospital of USTC, Division of Life Sciences and Medicine, University of Science and Technology of China, Hefei, 230001 Anhui China; 2 BGI Genomics, BGI-Shenzhen, Shenzhen, 518083 China; 3 BGI Education Center, University of Chinese Academy of Sciences, Shenzhen, 518083 China; 4 Dalian Municipal Women and Children’s Medical Center, Dalian, 116037 China

**Keywords:** Genetic testing, Genotype

## Abstract

Hearing loss is one of the most common birth disorders in humans, with an estimated prevalence of 1–3 in every 1000 newborns. This study investigates the molecular etiology of a hearing loss cohort using a stepwise strategy to effectively diagnose patients and address the challenges posed by the genetic heterogeneity and variable mutation spectrum of hearing loss. In order to target known pathogenic variants, multiplex PCR plus next-generation sequencing was applied in the first step; patients which did not receive a diagnosis from this were further referred for exome sequencing. A total of 92 unrelated patients with nonsyndromic hearing loss were enrolled in the study. In total, 64% (59/92) of the patients were molecularly diagnosed, 44 of them in the first step by multiplex PCR plus sequencing. Exome sequencing resulted in eleven diagnoses (23%, 11/48) and four probable diagnoses (8%, 4/48) among the 48 patients who were not diagnosed in the first step. The rate of secondary findings from exome sequencing in our cohort was 3% (2/58). This research presents a molecular diagnosis spectrum of 92 non-syndromic hearing loss patients and demonstrates the benefits of using a stepwise diagnostic approach in the genetic testing of nonsyndromic hearing loss.

## Introduction

Hearing loss is one of the most common birth defects in humans, with an estimated prevalence of 1–3 in every 1000 newborns^1^. Seventy percent of hearing loss cases are nonsyndromic, and one of the primary etiologies is genetic predisposition^1^. To date, over 100 genes have been associated with nonsyndromic hearing loss (NSHL)^2,3^, and new genes are continuing to be discovered^4^. The timely and effective diagnosis of affected individuals is made challenging by the extreme genetic heterogeneity underlying the condition.

Interestingly, the frequency of the genes behind NSHL varies between different populations and ethnicities. The most common mutations in many populations are in the *GJB2* gene, which encodes the connexin 26 protein, and cause severe-to-profound autosomal recessive NSHL^1,5^. Sanger sequencing of *GJB2* was therefore performed in previous studies^6,7^. In the Saudi population, the *OTOF* gene, rather than *GJB2*, was revealed to be a major and potential contributor to hearing loss^7^. *SLC26A4* is another common gene causing nonsyndromic hearing impairment with enlarged vestibular aqueducts in Asian and Middle Eastern populations and Ashkenazi Jews^8^. A single Sanger sequencing reaction is capable of covering the whole coding region of *GJB2* at an affordable cost as it has only 226 amino acids. *SLC26A4* has 21 exons and the coding sequencing spans 2343 bp from exon 2 to exon 21, requiring multiple Sanger sequencing reactions.

Although exome sequencing has been proposed and used as a single-step test for hearing loss patients^9,10^, interpreting exome sequencing data is usually laborious and time-consuming. Tiered or stepwise diagnostic approaches have been proposed multiple times in the literature^4,6,11,12^. Guan et al. provided a two-tier strategy which consisted of Sanger sequencing combined with targeted deletion analyses of *GJB2* and *STRC* and two mitochondrial genes, followed by exome sequencing and targeted analysis of deafness-related genes^6^. Li et al. performed hotspot variant screening and subsequent exome sequencing in a family with deafness^4^.

This study proposes a hierarchical approach that first targets known pathogenic variants by multiplex PCR, followed by exome sequencing and a comprehensive analysis of deafness genes. We have evaluated the performance of this strategy in a cohort of 92 NSHL patients. Individuals with inconclusive or negative results in the first step were referred for exome sequencing. An analysis of the diagnostic rate in the different steps and the contribution of different genetic factors enabled us to formulate a cost-effective diagnostic paradigm which can serve as an example for other populations.

## Results

Of the 92 NSHL patients, most (82%; 75/92) had no family history of hearing loss. The degree of hearing loss in the patients varied; severe-to-profound hearing loss was observed in the majority (84%, 77/92), and prelingual hearing loss was detected in 87% (80/92) patients. Of note, 17% (16/92) of the patients passed a newborn hearing screen at birth but developed hearing loss at a later age (Table 1).

**Table 1:** Characteristics of the study cohort^a^.

Characteristic	No. (%)
All	92 (100)
**Sex**	
Male	54 (59)
Female	38 (41)
**Family history**	
Yes	17 (18)
No	75 (82)
**Onset**	
Prelingual (≤ 3 years)	80 (87)
Post-lingual (> 3 years)	12 (13)
**Laterality**	
Bilateral symmetric	69 (75)
Bilateral asymmetric	17 (18)
No record	6 (7)
**Stability**	
Stable	58 (63)
Fluctuating	24 (26)
No record	10 (11)
**Aminoglycoside Exposure**	
Yes	4 (4)
No	63 (68)
Uncertain	25 (27)
**Severity**^b^	
Mild	1 (1)
Moderate	9 (10)
Severe	20 (22)
Profound	57 (62)
No record	5 (5)
**Rehabilitation**	
Hearing aid	41 (45)
Cochlear Implantation	17 (18)
Both	22 (24)
Not applied	12 (13)
**Newborn hearing screening result**	
Pass	16 (17)
Referral	36 (39)
Not applied/No record	40 (43)

^a^All members in this cohort have bilateral hearing loss; the precise type of hearing loss are not always recorded, most recorded cases are sensorineural; ^b^Severity is determined by the hearing level of the better ear; WHO grading rule is adopted (Mild: 26–40 dB; Moderate: 41–60 dB; Severe: 61–80 dB; Profound: > 80 dB).

### Forty-four diagnoses by multiplex PCR

**Figure 1:**
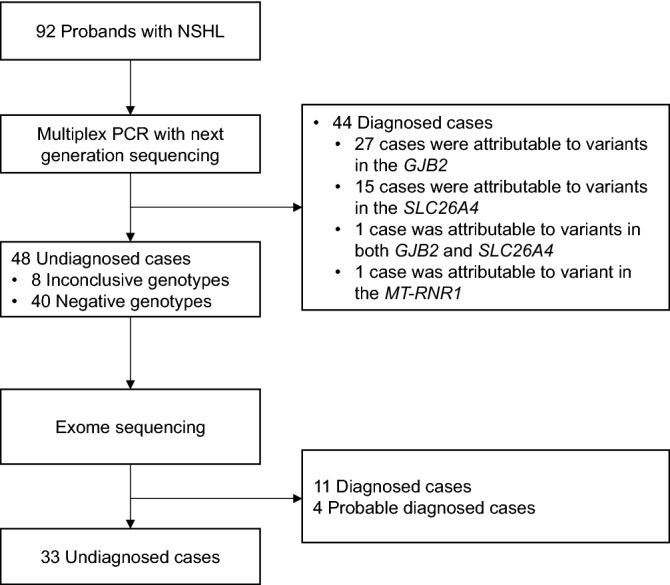
Outline of the study design. Ninety-two patients with non-syndromic hearing loss were enrolled. After carrying out multiplex PCR and next generation sequencing on all the patients, the 48 undiagnosed and 10 patients diagnosed for *GJB2* c.109G>A were referred for exome sequencing. *NSHL* non-syndromic hearing loss.

In the first step, the patients were tested via multiplex PCR. The tests yielded a positive result in 44 out of the 92 patients, while eight were inconclusive and 40 negative (Fig. 1). The genotypes of the 44 patients who tested positive are listed in Table 2. Classifications of these variants were presented in Table 3. There were 27 with a mutation in *GJB2*, 15 in *SLC26A4*, 1 with a dual molecular diagnosis of both *GJB2* and *SLC26A4*, and 1 with a mutation in *MT-RNR1*. The patient who was positive with a homoplasmic m.1555A>G in the *MT-RNR1* gene had aminoglycoside exposure history. Homozygous NM_004004.6:c.235delC in the *GJB2* gene was the most prevalent genotype, accounting for 11% (10/92) of the study cohort. NM_004004.6:c.109G>A in *GJB2* was identified in 10 of the 44 patients with positive genotypes, including three patients who were homozygous and seven patients who a compound heterozygous mutation. One patient was homozygous for both NM_000441.2:c.919-2A>G in *SLC26A4* and NM_004004.6:c.109G>A in *GJB2*. This patient was clinically diagnosed with deafness and enlarged vestibular aqueducts, a phenotype which can be caused by deficiency of the two genes together.

**Table 2:** Genotype of NSHL patients detected by Multiplex PCR sequencing.

No	Variant 1	Classification	Zygosity	Variant 2	Classification	Zygosity	Inheritance	Number of patients
**Diagnosed**								
1	*GJB2*(NM_004004.5): c.235delC(p.Leu79CysfsTer3)	Pathogenic	Hom	–	–	–	AR	10
2	*GJB2*(NM_004004.5): c.235delC(p.Leu79CysfsTer3)	Pathogenic	Het	*GJB2*(NM_004004.5): c.299_300delAT(p.His100ArgfsTer14)	Pathogenic	Het	AR	3
3	*GJB2*(NM_004004.5): c.235delC(p.Leu79CysfsTer3)	Pathogenic	Het	*GJB2*(NM_004004.5): c.176_191del(p.Gly59AlafsTer18)	Pathogenic	Het	AR	3
4	*GJB2*(NM_004004.5): c.235delC(p.Leu79CysfsTer3)	Pathogenic	Het	*GJB2*(NM_004004.5): c.109G>A(p.Val37Ile)	Pathogenic	Het	AR	3
5	*GJB2*(NM_004004.5): c.109G>A(p.Val37Ile)	Pathogenic	Hom	–	–	–	AR	3
6	*SLC26A4*(NM_000441.1): c.919-2A>G	Pathogenic	Hom	–	–	–	AR	2
7	*SLC26A4*(NM_000441.1): c.919-2A>G	Pathogenic	Hom	*GJB2*(NM_004004.5): c.109G>A(p.Val37Ile)	Pathogenic	Hom	AR	1
8	*GJB2*(NM_004004.5): c.176_191del(p.Gly59AlafsTer18)	Pathogenic	Het	*GJB2*(NM_004004.5): c.299_300delAT(p.His100ArgfsTer14)	Pathogenic	Het	AR	1
9	*GJB2*(NM_004004.5): c.299_300delAT(p.His100ArgfsTer14)	Pathogenic	Het	*GJB2*(NM_004004.5): c.109G>A(p.Val37Ile)	Pathogenic	Het	AR	1
10	*GJB2*(NM_004004.5): c.109G>A(p.Val37Ile)	Pathogenic	Het	*GJB2*(NM_004004.5): c.427C>T(p.Arg143Trp)	Pathogenic	Het	AR	1
11	*GJB2*(NM_004004.5): c.109G>A(p.Val37Ile)	Pathogenic	Het	*GJB2*(NM_004004.5): c.428G>A(p.Arg143Gln)	Pathogenic	Het	AR	1
12	*GJB2*(NM_004004.5): c.109G>A(p.Val37Ile)	Pathogenic	Het	*GJB2*(NM_004004.5): c.583A>G(p.Met195Val)	Likely pathogenic	Het	AR	1
13	*SLC26A4*(NM_000441.1): c.919-2A>G	Pathogenic	Het	*SLC26A4*(NM_000441.1): c.1174A>T(p.Asn392Tyr)	Pathogenic	Het	AR	1
14	*SLC26A4*(NM_000441.1): c.1226G>A(p.Arg409His)	Pathogenic	Het	*SLC26A4*(NM_000441.1): c.2000T>C(p.Phe667Ser)	Pathogenic	Het	AR	1
15	*SLC26A4*(NM_000441.1): c.1229C>T(p.Thr410Met)	Pathogenic	Het	*SLC26A4*(NM_000441.1): c.1343C>A(p.Ser448Ter)	Pathogenic	Het	AR	1
16	*SLC26A4*(NM_000441.1): c.1229C>T(p.Thr410Met)	Pathogenic	Het	*SLC26A4*(NM_000441.1): c.1692dupA(p.Cys565MetfsTer9)	Pathogenic	Het	AR	1
17	*SLC26A4*(NM_000441.1): c.1229C>T(p.Thr410Met)	Pathogenic	Het	*SLC26A4*(NM_000441.1): c.1707 + 5G>A	Pathogenic	Het	AR	1
18	*SLC26A4*(NM_000441.1): c.1229C>T(p.Thr410Met)	Pathogenic	Het	*SLC26A4*(NM_000441.1): c.919-2A>G	Pathogenic	Het	AR	1
19	*SLC26A4*(NM_000441.1): c.1336C>T(p.Gln446Ter)	Pathogenic	Het	*SLC26A4*(NM_000441.1): c.919-2A>G	Pathogenic	Het	AR	1
20	*SLC26A4*(NM_000441.1): c.1343C>T(p.Ser448Leu)	Pathogenic	Het	*SLC26A4*(NM_000441.1): c.2168A>G(p.His723Arg)	Pathogenic	Het	AR	1
21	*SLC26A4*(NM_000441.1): c.2000T>C(p.Phe667Ser)	Pathogenic	Het	*SLC26A4*(NM_000441.1): c.2168A>G(p.His723Arg)	Pathogenic	Het	AR	1
22	*SLC26A4*(NM_000441.1): c.589G>A(p.Gly197Arg)	Pathogenic	Het	*SLC26A4*(NM_000441.1): c.2168A>G(p.His723Arg)	Pathogenic	Het	AR	1
23	*SLC26A4*(NM_000441.1): c.919-2A>G	Pathogenic	Het	*SLC26A4*(NM_000441.1): c.1991C>T(p.Ala664Val)	Pathogenic	Het	AR	1
24	*SLC26A4*(NM_000441.1): c.919-2A>G	Pathogenic	Het	*SLC26A4*(NM_000441.1): c.1614 + 1G>A	Pathogenic	Het	AR	1
25	*SLC26A4*(NM_000441.1): c.919-2A>G	Pathogenic	Het	*SLC26A4*(NM_000441.1): c.668T>C(p.Phe223Ser)	Pathogenic	Het	AR	1
26	*MT-RNR1*: m.1555A>G	Pathogenic	Homo	–	–	–	Mi	1
**Inconclusive**								
27	*GJB2*(NM_004004.5): c.109G>A(p.Val37Ile)	Pathogenic	Het	–	–	–	AR	3
28	*GJB2*(NM_004004.5): c.235delC(p.Leu79CysfsTer3)	Pathogenic	Het	–	–	–	AR	2
29	*SLC26A4*(NM_000441.1): c.919-2A>G	Pathogenic	Het	–	–	–	AR	1
30	*SLC26A4*(NM_000441.1): c.2168A>G(p.His723Arg)	Pathogenic	Het	–	–	–	AR	1
31	*SLC26A4*(NM_000441.1): c.1229C>T(p.Thr410Met)	Pathogenic	Het	–	–	–	AR	1

*Hom* homozygous, *Het* heterozygous, *Homo* homoplasmy, *AR* autosomal recessive, *AD* autosomal dominant, *Mi* mitochondrial.

**Table 3:** Classification of variants detected in first step.

No	Variant	Classification	Criteria applied	Reference PubMed ID
1	*GJB2*(NM_004004.5):c.109G>A(p.Val37Ile)	Pathogenic	PS4, PM1, PM3, PP1_Strong, BS2	31160754
2	*GJB2*(NM_004004.5):c.176_191del(p.Gly59AlafsTer18)	Pathogenic	PVS1, PS3_Moderate, PM2, PM3_VeryStrong	20095872; 26043044
3	*GJB2*(NM_004004.5):c.235delC(p.Leu79CysfsTer3)	Pathogenic	PVS1, PS3_Moderate, PM3_VeryStrong	12352684; 26043044; 1245676
4	*GJB2*(NM_004004.5):c.299_300delAT(p.His100ArgfsTer14)	Pathogenic	PVS1, PS3_Moderate, PM2_Supporting, PM3_VeryStrong	20095872; 26043044
5	*GJB2*(NM_004004.5):c.427C>T(p.Arg143Trp)	Pathogenic	PM2_Supporting, PM3_VeryStrong, PM5, PP3	27792752; 28271504; 26095810; 24256046; 14985372
6	*GJB2*(NM_004004.5):c.428G>A(p.Arg143Gln)	Pathogenic	PM2, PM3_VeryStrong, PM5, PP3	23856378; 19715472; 22991996; 11313763
7	*GJB2*(NM_004004.5):c.583A>G(p.Met195Val)	Likely pathogenic	PM2_Supporting, PM3_Strong, PP3	20497192; 24507663; 24013081
8	*SLC26A4*(NM_000441.1):c.1174A>T(p.Asn392Tyr)	Pathogenic	PM2, PM3_VeryStrong, PP3, PP4	23151025; 28786104; 24599119
9	*SLC26A4*(NM_000441.1):c.1226G>A(p.Arg409His)	Pathogenic	PM1, PM2_Supporting, PM3_VeryStrong, PP3, PP4	27247933; 28786104; 25372295
10	*SLC26A4*(NM_000441.1):c.1229C>T(p.Thr410Met)	Pathogenic	PS4, PM1, PM2_Supporting, PM3_VeryStrong, PM5, PP3, PP4	23151025; 28786104; 23638949
11	*SLC26A4*(NM_000441.1):c.1336C>T(p.Gln446Ter)	Pathogenic	PVS1, PM2, PM3	25372295
12	*SLC26A4*(NM_000441.1):c.1343C>A(p.Ser448Ter)	Pathogenic	PVS1, PM2, PM3, PP4	25372295
13	*SLC26A4*(NM_000441.1):c.1343C>T(p.Ser448Leu)	Pathogenic	PM2, PM3_VeryStrong, PP3, PP4	21961810; 25372295; 24599119; 24612839
14	*SLC26A4*(NM_000441.1):c.1614 + 1G>A	Pathogenic	PVS1, PM2, PM3_Strong, PP4	11919333; 25372295; 20128824
15	*SLC26A4*(NM_000441.1):c.1692dupA(p.Cys565MetfsTer9)	Pathogenic	PVS1, PM2, PM3, PP4	25372295
16	*SLC26A4*(NM_000441.1):c.1707 + 5G>A	Pathogenic	PS3_VeryStrong, PS4, PM2, PM3_VeryStrong, PP3, PP4	24599119; 31599023
17	*SLC26A4*(NM_000441.1):c.1991C>T(p.Ala664Val)	Pathogenic	PM2, PM3_VeryStrong, PP3, PP4	25372295
18	*SLC26A4*(NM_000441.1):c.2000T>C(p.Phe667Ser)	Pathogenic	PM2, PM3_Strong, PM5, PP3, PP4	22412181; 25372295
19	*SLC26A4*(NM_000441.1):c.2168A>G(p.His723Arg)	Pathogenic	PS3_Supporting, PM3_VeryStrong, PP3, PP4, BS1_Supporting	18310264; 25372295
20	*SLC26A4*(NM_000441.1):c.589G>A(p.Gly197Arg)	Pathogenic	PM2, PM3_VeryStrong, PP1, PP3, PP4	25372295; 23385134
21	*SLC26A4*(NM_000441.1):c.668T>C(p.Phe223Ser)	Pathogenic	PM2, PM3_VeryStrong, PP3, PP4	25372295; 30554688
22	*SLC26A4*(NM_000441.1):c.919-2A>G	Pathogenic	PVS1, PS4, PM3_VeryStrong, PP4, BS1	25149764; 23638949
23	*MT-RNR1*(NC_012920.1):m.1555A>G	Pathogenic	–	31160754

### Fifteen diagnoses/probable diagnoses by exome sequencing

Two groups of patients (n = 58) were referred for exome sequencing. Group 1 was the 48 patients who received inconclusive or negative genotypes from the multiplex PCR (Fig. 1). Group 2 consisted of 10 patients who were either homozygous or compound heterozygous for NM_004004.6:c.109G>A in *GJB2*. Due to the variable expressivity and incomplete penetrance of NM_004004.6:c.109G>A in *GJB2*^13^, these 10 patients were referred for exome sequencing in order to exclude other potential molecular etiologies.

In the first group, exome sequencing resulted in eleven diagnoses (23%, 11/48) and four probable diagnoses (8%, 4/48) (Table 4). No other causally associated variants related to hearing loss were identified in the second group. It is worth noting that patient P27 was first identified as homozygous for NM_001038603.3(*MARVELD2*):c.1208_1211delGACA by exome sequencing*.* Given that the family history did not indicate consanguinity, we performed CNV analysis of the exome data from this patient as well as all the other fifty-seven patients in step 2. The exome data revealed a heterozygous deletion of exon 3 to exon 5 in the *MARVELD2* gene, which was also verified by qPCR. Variant c.1208_1211delGACA is located in exon 4, which was deleted in this CNV variant. We thus conclude that c.1208_1211delGACA is hemizygous in this case.

**Table 4:** Diagnoses solely made by exome sequencing.

Patient ID	Gene transcript	Variant	Zygosity	Classification	Criteria applied	Reference PubMed ID	Inheritance	Onset	Severity	Family history
P11	*SLC26A4* NM_000441.1	c.1229C>T (p.Thr410Met)#	Het	Pathogenic	PS4, PM1, PM2_Supporting, PM3_VeryStrong, PM5, PP3, PP4	23151025; 28786104; 23638949	AR	Post-lingual	Profound	YES
		c.164 + 1G>C	Het	Pathogenic	PVS1, PM2, PM3	25724631				
P15	*COL11A2* NM_080680.2	c.966_967insC (p.Thr323Hisfs*19)	Het	Pathogenic	PVS1, PM2, PM3_Supporting	29456477	AR	Prelingual	Severe	NO
		c.1879C>T (p.Arg627*)	Het	Likely pathogenic	PVS1, PM2	Novel				
P27	*MARVELD2* NM_001038603.2	c.1208_1211del (p.Arg403Lysfs*11)	Hemi	Likely pathogenic	PVS1, PM2	Novel	AR	Post-lingual	Profound	NO
		EX3_EX5 DEL	Het	Likely pathogenic	PVS1, PM2	Novel				
P35	*MYO15A* NM_016239.3	c.8791delT (p.Trp2931Glyfs*103)	Het	Pathogenic	PVS1, PM2, PM3_Supporting	30953472; 23767834	AR	Prelingual	Profound	NO
		c.10419_10423del (p.Ser3474Profs*42)	Het	Pathogenic	PVS1_Moderate, PM2, PM3	10.15761/OHNS.1000207				
P44	*MITF* NM_000248.3	c.763C>T (p.Arg255*)	Het	Pathogenic	PVS1, PS3_Supporting, PM2, PP1, PP4	24194866; 29094203; 29531335	AD	Prelingual	Profound	NO
P58	*MYO15A* NM_016239.3	c.7308delA (p.Arg2436Serfs*34)	Het	Likely pathogenic	PVS1, PM2	Novel	AR	Prelingual	Profound	NO
		c.9690 + 1G>A	Het	Pathogenic	PVS1, PM2, PM3	29849560				
P59	*CDH23* NM_022124.5	c.9389_9390delCT (p.Pro3130Argfs*19)	Hom	Pathogenic	PVS1, PM2, PM3	29568747	AR	Prelingual	Profound	NO
P76	*SLC26A4* NM_000441.1	c.919-2A>G#	Het	Pathogenic	PVS1, PS4, PM3_VeryStrong, PP4, BS1	25149764; 23638949	AR	Prelingual	Severe	NO
		c.916dupG (p.Val306Glyfs*24)	Het	Pathogenic	PVS1, PM2, PM3, PP4	26252218; 17718863				
P97	*COL11A2* NM_080680.2	c.4135C>T (p.Arg1379*)	Het	Pathogenic	PVS1, PM2, PP1	15372529	AD	Post-lingual	No record	YES
P104	*MYO15A* NM_016239.3	c.4039-2A>C	Het	Likely pathogenic	PVS1, PM2	Novel	AR	Prelingual	Profound	NO
		c.7720C>T (p.Gln2574*)	Het	Pathogenic	PVS1, PM2, PP1_Strong	30943474				
P111	*SOX10* NM_006941.3	c.482G>A (p.Arg161His)	Het	Likely Pathogenic	PS2, PS3_Supporting, PM2, PP4	31152317; 21898658; 28000701	AD	Prelingual	Profound	NO
**Probable diagnoses**										
P52	*OTOA* NM_144672.3	c.1352G>A (p.Gly451Asp)	Het	Likely pathogenic	PM2, PP1_Strong, PP3	23173898	AR	Prelingual	Moderate	NO
		c.1265G>T (p.Gly422Val)	Het	VUS	PM2_Supporting	Novel				
P63	*TRIOBP* NM_001039141.2	c.4919A>G (p.Asn1640Ser)	Het	VUS	PM2_Supporting, BP4	Novel	AR	Prelingual	Severe	YES
		c.5185-2A>G	Het	Likely pathogenic	PVS1, PM2	Novel				
P89	*TMPRSS3* NM_024022.2	c.551T>C (p.Leu184Ser)	Het	Likely Pathogenic	PS3_Supporting, PM2_Supporting, PM3_Strong, PP1	31379920; 31016883; 32235586	AR	Prelingual	Profound	NO
		c.235T>C (p.Cys79Arg)	Het	VUS	PM2, PP3	Novel				
P102	*BSND* NM_057176.2	c.88C>T (p.Arg30Trp)	Het	VUS	PM2	Novel	AR	Prelingual	Profound	NO
		c.318delC (p.Tyr107MetfsTer13)	Het	Likely pathogenic	PVS1, PM2	Novel				

All patients received a negative or inconclusive result in the multiplex PCR test. *Hom* homozygous, *Het* heterozygous, *Hemi* hemizygous, *AR* autosomal recessive, *AD* autosomal dominant. ^#^Variants also detected by multiplex PCR; Patient P63 is also a carrier of NM_004004.5(*GJB2*):c.235delC; Patient P89 is also a carrier of NM_000441.1(*SLC26A4*):c.2168A>G.

**Figure 2:**
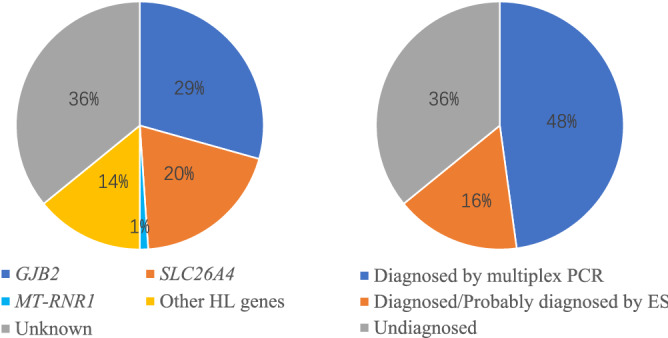
Genetic spectrum of enrolled non-syndromic hearing loss probands. Molecular diagnostic results were classified according to genes and detection methods. *ES* exome sequencing.

Summing up, the two-step approach identified the molecular etiology of 59/92 (64%) NSHL index patients, including 44 (48%) diagnoses by multiplex PCR in the first step and 15 (16%) diagnoses/probable diagnoses by exome sequencing (Fig. 2). Mutations in *GJB2* were the most frequent (28/59), followed by mutations in *SLC26A4* (18/59), *MYO15A* (3/59), *COL11A2* (2/59), and *MT-RNR1, MARVELD2, MITF, CDH23, OTOA, TRIOBP, TMPRSS3, SOX10* and *BSND* (1/59 each).

Further analysis of the exome sequencing data was carried out to discover secondary findings. The cohort had two pathogenic variants from among the 59 secondary findings genes recommended by the ACMG^14^ (Supplementary Table S2). A heterozygous variant, *GLA*(NM_000169.3):c.1067G>A (p.Arg356Gln) was identified in patient P21 (female). It is related to Fabry disease, an X-linked inborn error of glycosphingolipid catabolism resulting from deficient or absent activity of the lysosomal enzyme alpha-galactosidase A^15^. The second pathogenic variant was *RYR1*(NM_000540.3):c.6502G>A (p.Val2168Met), which is associated with malignant hyperthermia. This variant was identified in P77 in a heterozygous state. The rate of secondary findings in our cohort was 3% (2/58), comparable to previously published rates of 1.8% to 4.6%^16–19^.

## Discussion

This study applied a stepwise, genetic testing approach to explore molecular diagnoses in an NSHL cohort, achieving 64% (59/92) diagnostic yield. Although diagnostic yield varies between different patient cohorts and depends on the detection methods used, our diagnostic yield (64%) is comparable to a multi-ethnic cohort tested using exome sequencing (56%)^9^.

This study uncovered the etiology of 44 patients in the first step using a commercial multiplex PCR kit, providing a rapid molecular diagnosis and saving the cost of exome sequencing. The multiplex PCR contains amplicons covering the *GJB2, SLC26A4* and *MT-RNR1* genes. These three genes are known to have hotspot variants causing non-syndromic hearing loss in Asian populations, including c.109G>A, c.235delC, and c.299_300delAT in *GJB2*, c.919-2A>G, c.1229C>T, and c.2168A>G in *SLC26A4*, and m.1555A>G in *MT-RNR1*^20–22^. Compared to a single test of *GJB2*, which is frequently used as the first-tier test to exclude hotspot variants before exome sequencing^6,9^, a multiplex PCR sequencing approach appears to be both efficient and cost-effective, as it is more flexible and can detect hotspot variants across multiple deafness-related genes.

It should be noted that the diagnostic rate of the multiplex PCR assay in the first step could vary dramatically in different populations depending on the prevalence of hotspot variants in targeted patients. In this study, the high diagnostic rate was attributable to the enrichment of NM_004004.6(*GJB2*):c.109G>A, NM_004004.6(*GJB2*):c.235delC, NM_004004.6(*GJB2*):c.299_300delAT, NM_000441.2(*SLC26A4*):c.919-2A>G, and NM_000441.2(*SLC26A4*): c.1229C>T in the Chinese population^23,24^. More importantly, ethnic background is relatively uniform in China. The inherent ethnic bias of the multiplex PCR assay is a drawback and might be inappropriate in a racially and ethnically diverse population^25^.

Allelic heterogeneity is common in hearing loss and is associated with clinical phenotype heterogeneity, with both syndromic hearing loss and NSHL being caused by mutations within the same gene^26^. In this study, although we only enrolled patients with nonsyndromic hearing loss, deafness-related variants were also identified in syndromic genes. P44 was heterozygous for a disease-causing nonsense variant in the *MITF* gene, and P111 was heterozygous for a disease-causing missense variant in the *SOX10* gene. Both variants are associated with autosomal dominant inherited Waardenburg syndrome, which is considered an NSHL mimic. The variability of phenotypes makes clinical diagnosis and variant interpretation in genetic hearing loss challenging^27^.

The molecular diagnosis of NSHL is made yet more challenging by the variable expressivity and high prevalence of NM_004004.6:c.109G>A in the *GJB2* gene^13^. In our cohort, one patient with enlarged vestibular aqueducts was found to be homozygous for both NM_000441.2:c.919-2A>G in *SLC26A4* and NM_004004.6:c.109G>A in *GJB2* in the first diagnosis step. The genotype–phenotype consistency led us to consider NM_000441.2:c.919-2A>G in *SLC26A4* as the disease-causing variant. However, we cannot rule out the possibility of a blended phenotype arising from the two variants^28^. By contrast, 10 patients who were diagnosed with only NM_004004.6:c.109G>A in *GJB2* in the first step were referred for exome sequencing, and no other potential molecular explanations were identified. These results indicate the importance of incorporating phenotype and genotype in practice and of considering dual molecular diagnoses.

Copy number variations are common causes of nonsyndromic hearing loss^29^. Exome sequencing data can be analyzed for CNVs, although such analyses suffer from low sensitivity and uncertain specificity^30^. In this study, one patient in our cohort was diagnosed to have a SNV compounded with a CNV in *MARVELD2*. The homozygous c.1208_1211delGACA(p.Arg403Lysfs*11) in the *MARVELD2* gene was initially thought to be the causal etiology*.* The lack of consanguineous history led us to reanalyze the coverage depth of the exons in the *MARVELD2* gene, resulting in the identification of an EX3_EX5 Del. This finding highlights the importance of considering CNV deletions in a non-consanguineous family when a pathogenic variant is identified in a homozygous state.

It is worth noting that 17% (16/92) of the patients passed a newborn hearing screening at birth but developed hearing loss at a later stage. Seven of these patients received a positive molecular diagnosis, with variants in the *GJB2* and *SLC26A4* genes (Supplementary Table S3). These results are consistent with recent findings showing that newborns with positive genotypes can be missed by physiologic newborn hearing screens but identified by genetic screens, highlighting the necessity of concurrent hearing and genetic screening in newborns^21,31^.

This study has several limitations which should be noted. First, the stepped approach may miss a dual molecular diagnosis if a patient is diagnosed in the first step. A dual molecular diagnosis was reported in 4.9% of patients with multiple phenotypic traits^28^. For single phenotype traits such as NSHL, dual molecular diagnosis might be rare, but this is worth considering when deciding on a diagnostic approach. Second, CNV analysis of the *STRC* gene is absent in our exome data pipelines due to the presence of a pseudogene. This might result in an underestimate of the contribution of disease-causing CNVs in this study.

In conclusion, this work demonstrates the benefits of a stepwise approach to diagnose non-syndromic hearing loss patients. Instead of starting with exome sequencing, multiplex PCR targeted hotspot variants across multiple genes can provide a molecular etiology for 48% of Eastern Asian patients in a prompt and efficient manner. It seems likely that this will result in significant savings, but a future cost-effectiveness analysis will address that question.

## Methods

### Participants

A total of 92 patients with NSHL in Eastern Asian ethnicity were recruited. No other visible phenotype was reported. We obtained informed consent from the patients. This study was approved by the Institutional Review Board of BGI. All methods were performed in accordance with the relevant guidelines and regulations.

### Study design

The enrolled patients were first assayed with a commercial multiplex PCR to analyze common variants in the Asian population. Patients undiagnosed by the multiplex PCR assay were then referred for exome sequencing. Because of the variable expressivity and penetrance of NM_004004.6:c.109G>A in *GJB2*, patients diagnosed with this variant were also referred for exome sequencing to explore other potential molecular etiologies.

### Multiplex PCR

Genetic variant detection was carried out in all patients by applying multiplex PCR combined with next-generation sequencing. The commercial multiplex PCR kit (BGI Biotech, Wuhan, China) was designed to cover certain pathogenic variants of 22 genes, including the complete coding region of *GJB2* and most of the coding regions of *SLC26A4*. Genomic DNA was extracted from 2 ml of peripheral blood using a DNA Extraction Kit (BGI Biotech, Wuhan, China). Targeted variants were amplified by multiplex PCR using 2 × KAPA 2G Fast Multiple PCR Mix (KAPA BIOSYSTEMS, Wilmington, MA, USA). The PCR program consisted of one round of 95 °C for 3 min, then 30 cycles of 95 °C for 15 s, 62 °C for 30 s, and 72 °C for 90 s.

### Library preparation, sequencing and bioinformatics

PCR products were pooled to prepare a library. Briefly, ~ 3.5 μg purified products were sheared by ultrasonoscope and quality-controlled using an Agilent Bioanalyzer DNA 2100 kit (Agilent, Santa Clara, CA, USA). Subsequently, end-repair and A-tailing were performed before adapters were ligated to both ends of the fragments. Finally, the adapter-ligated products were amplified by 8-cycle PCR and purified using Agencourt AMPure XP beads (Beckman Coulter, Fullerton, CA, USA). The prepared libraries were subjected to single-strand circularized DNA and DNA nanoball preparation before being sequenced on a BGISEQ-500 sequencer (BGI, Shenzhen, China) with PE50^32^. Raw sequence reads were mapped to the human reference genome (hg19) using Bowtie 2.3.3 with SAMtools 1.6 used to create BAM and index files. For variant calling, Genome Analysis Tool Kit (GATK 3.7)^33^ was used to analyze the alignment data.

### Exome sequencing and data analysis

Exome sequencing was performed following standard manufacturer protocols on a BGISEQ-500 platform. An in-house bioinformatics pipeline was employed to process the variant call format (VCF) files and to maintain variants of potential clinical usefulness, including (i) variants with minor allele frequency (MAF) < 1%, (ii) variants in genes with an OMIM disease entry. We interpreted variants in 130 genes curated by ClinGen Expert as having a limited-to-definitive relationship to hearing loss^34^. This interpretation was based on ClinGen Expert Specification of the ACMG/AMP Variant Interpretation Guidelines for Genetic Hearing Loss^27^.

### Definition of molecular diagnosis

 Patients were categorized as “positive” or “diagnosed” if they were homozygous or double heterozygous for a pathogenic/likely pathogenic variant(s) in a recessive inherited gene or heterozygous for a pathogenic/likely pathogenic variant in a dominant inherited gene. In addition, patients with a pathogenic/likely pathogenic variant plus a rare VUS in a recessive inherited gene were considered “probably diagnosed.” Patients with a pathogenic/likely pathogenic variant in a recessive inherited gene were considered “inconclusive”. Patients with a variant in a gene that was not inherited recessively or dominantly, for example in mitochondrial genes, were categorized as “diagnosed” if the phenotype associated with the genotype.

### Sanger validation and qPCR

Sanger sequencing was carried out to validate SNPs/Indels detected by either multiplex PCR or exome sequencing. All PCR products were sequenced on an ABI 3730XL DNA Analyzer. Mutations were confirmed by comparing our sequencing data with the UCSC human reference sequences^35^. Verification of exon-level deletions or duplications called by exome sequencing was carried out by qPCR. The qPCR methodology has been previously described^36^. The primer pair sequences are shown in Supplementary Table S1.

## Supplementary Information

Supplementary Tables.
