# Supplementary material for: Molecular diagnosis of non-syndromic hearing loss patients using a stepwise approach
Source: Sci Rep. 2021 Feb 17;11:4036. doi: 10.1038/s41598-021-83493-6 (PMC7889619; doi:10.1038/s41598-021-83493-6)
Supplement: Supplementary file 1 — Supplementary Tables. [file 41598_2021_83493_MOESM1_ESM.docx]

Molecular diagnosis of non-syndromic hearing loss patients using a stepwise approach

Running title: Stepwise diagnosis of hearing loss patients

Jing Wang^1^, Jiale Xiang^2,3^, Lisha Chen^2,3^, Hongyu Luo^2^, Xiuhua Xu^4^, Nan Li^4^, Chunming Cui^4^, Jingjing Xu^1^, Nana Song^2^, Jiguang Peng^2^, Zhiyu Peng^2*^

Jing Wang, Jiale Xiang, Lisha Chen contributed equally to this work.

*Correspondence to Dr. Zhiyu Peng at pengzhiyu@bgi.com and +86 075536307011

BGI Park, No.21 Hongan 3rd Street, Yantian District, Shenzhen 518083, China

1. Department of Obstetrics and Gynecology, The First Affiliated Hospital of USTC, Division of Life Sciences and Medicine, University of Science and Technology of China, Hefei, Anhui 230001, China.

2. BGI Genomics, BGI-Shenzhen, Shenzhen 518083, China

3. BGI Education Center, University of Chinese Academy of Sciences, Shenzhen 518083, China

4. Dalian municipal women and children's medical center, Dalian 116037, China

**Table S1. The primer pair sequences for qPCR**

| Exons | Sequence |
| --- | --- |
| MARVELD2-IN3-Forward | 5’- TGAAGTCTGTGCTTTAAGTGCTG -3’ |
| MARVELD2-IN3-Reverse | 3’- TGCCCACATTAGATTCCGGC -5’ |
| MARVELD2-EX5-Forward | 5’- GTGTTCCAAGACCAGTTTTCAG -3’ |
| MARVELD2-EX5-Reverse | 3’- ATCACTGCATCCAGCTCATCAA -5’ |

**Table S2. Secondary findings in patients**

| Patient ID | Gender | Gene | Transcript | Variant | Zygosity | Inheritance | Classification | Disease | Reference |
| --- | --- | --- | --- | --- | --- | --- | --- | --- | --- |
| P21 | Female | *GLA* | NM_000169.2 | c.1067G>A(p.Arg356Gln) | Het | XL | Pathogenic | Fabry disease | PMID: 28615118; 31996269 |
| P77 | Female | *RYR1* | NM_000540.2 | c.6502G>A(p.Val2168Met) | Het | AD | Pathogenic | Malignant hyperthermia susceptibility | PMID: 9497245; 11668625 |

Het, heterozygous; XL, X-linked; AD, autosomal dominant.

**Table S3. Auditory phenotypes and genotypes of patients passed newborn hearing screening**

| Patient ID | Onset  of HL | Hearing（dB） | | Severity | Stability | Genotype |
| --- | --- | --- | --- | --- | --- | --- |
|  |  | Right | Left |  |  |  |
| P7 | Post-lingual | 70 | 85 | Severe | Stable | - |
| P14 | Prelingual | 110 | 90 | Profound | Fluctuating | *GJB2*(NM_004004.6):c.235delC(;)299_300delAT |
| P21 | Post-lingual | 105 | 105 | Profound | Stable | - |
| P22 | Prelingual | 65 | 70 | Severe | Fluctuating | *GJB2*(NM_004004.6): c.109G>A(;)109G>A |
| P32 | Prelingual | 105 | 105 | Profound | Fluctuating | - |
| P33 | Prelingual | 100 | 100 | Profound | Stable | *GJB2*(NM_004004.6): c.235delC(;)235delC |
| P51 | Post-lingual | 85 | 85 | Profound | Stable | - |
| P54 | Prelingual | 95 | 95 | Profound | Stable | *SLC26A4*(NM_000441.2): c.668T>C(;)919-2A>G |
| P60 | Prelingual | 110 | 110 | Profound | Stable | - |
| P66 | Prelingual | 90 | 80 | Severe | Stable | - |
| P67 | Prelingual | 95 | 85 | Profound | Stable | *GJB2*(NM_004004.6): c.427C>T(;)109G>A; |
| P80 | Prelingual | 70 | 97 | Severe | Stable | - |
| P87 | Post-lingual | 55 | 120 | Moderate | Stable | - |
| P88 | Prelingual | 90 | 105 | Profound | Fluctuating | - |
| P95 | Prelingual | 90 | 80 | Profound | Stable | *SLC26A4*(NM_000441.2):c.919-2A>G(;)919-2A>G |
| P103 | Post-lingual | 100 | 110 | Profound | Fluctuating | *SLC26A4*(NM_000441.2):c.1229C>T(;)919-2A>G |

**Table S4. Variants covered by commercial multiplex PCR kit.**

| No. | Gene | Variant |  | No. | Gene | Variant |
| --- | --- | --- | --- | --- | --- | --- |
| 1 | *CDH23* | c.902G>A |  | 41 | *GJB2* | c.283G>A |
| 2 | *COL11A1* | c.4171-2A>G |  | 42 | *GJB2* | c.290dupA |
| 3 | *DSPP* | c.52G>T |  | 43 | *GJB2* | c.298C>T |
| 4 | *GJB2* | c.34G>C |  | 44 | *GJB2* | c.299_300delAT |
| 5 | *GJB2* | c.35delG |  | 45 | *GJB2* | c.310_323del |
| 6 | *GJB2* | c.35dupG |  | 46 | *GJB2* | c.313_326del |
| 7 | *GJB2* | c.35G>A |  | 47 | *GJB2* | c.365A>T |
| 8 | *GJB2* | c.35G>T |  | 48 | *GJB2* | c.370C>T |
| 9 | *GJB2* | c.44A>C |  | 49 | *GJB2* | c.427C>T |
| 10 | *GJB2* | c.50C>T |  | 50 | *GJB2* | c.428G>A |
| 11 | *GJB2* | c.71G>A |  | 51 | *GJB2* | c.439G>A |
| 12 | *GJB2* | c.94C>T |  | 52 | *GJB2* | c.487A>C |
| 13 | *GJB2* | c.95G>A |  | 53 | *GJB2* | c.487A>G |
| 14 | *GJB2* | c.95G>T |  | 54 | *GJB2* | c.504_505insAAGG |
| 15 | *GJB2* | c.99delT |  | 55 | *GJB2* | c.506G>A |
| 16 | *GJB2* | c.104T>G |  | 56 | *GJB2* | c.508_511dupAACG |
| 17 | *GJB2* | c.107T>C |  | 57 | *GJB2* | c.509dupA |
| 18 | *GJB2* | c.109G>A |  | 58 | *GJB2* | c.512_513insAACG |
| 19 | *GJB2* | c.132G>A |  | 59 | *GJB2* | c.535G>A |
| 20 | *GJB2* | c.132G>C |  | 60 | *GJB2* | c.551G>A |
| 21 | *GJB2* | c.134G>A |  | 61 | *GJB2* | c.551G>C |
| 22 | *GJB2* | c.139G>T |  | 62 | *GJB2* | c.564_565delGA |
| 23 | *GJB2* | c.155_158delTCTG |  | 63 | *GJB2* | c.575_576delCA |
| 24 | *GJB2* | c.164C>A |  | 64 | *GJB2* | c.576delA |
| 25 | *GJB2* | c.169C>T |  | 65 | *GJB2* | c.583A>G |
| 26 | *GJB2* | c.175G>A |  | 66 | *GJB2* | c.596C>T |
| 27 | *GJB2* | c.176_191del |  | 67 | *GJB2* | c.598G>T |
| 28 | *GJB2* | c.187G>T |  | 68 | *GJB2* | c.605G>T |
| 29 | *GJB2* | c.224G>A |  | 69 | *GJB2* | c.632_633delGT |
| 30 | *GJB2* | c.229T>C |  | 70 | *GJB3* | c.538C>T |
| 31 | *GJB2* | c.230G>A |  | 71 | *GSDME* | c.1183+4A>G |
| 32 | *GJB2* | c.231G>A |  | 72 | *KCNJ10* | c.491C>T |
| 33 | *GJB2* | c.235delC |  | 73 | *MT-RNR1* | m.1494C>T |
| 34 | *GJB2* | c.238C>T |  | 74 | *MT-RNR1* | m.1555A>G |
| 35 | *GJB2* | c.250G>A |  | 75 | *MT-TL1* | m.3243A>G |
| 36 | *GJB2* | c.250G>C |  | 76 | *MT-TS1* | m.7445A>G |
| 37 | *GJB2* | c.257C>G |  | 77 | *MYO15A* | c.8183G>A |
| 38 | *GJB2* | c.269dupT |  | 78 | *MYO15A* | c.8767C>T |
| 39 | *GJB2* | c.269T>C |  | 79 | *MYO7A* | c.133-2A>G |
| 40 | *GJB2* | c.280_284dupCACGT |  | 80 | *MYO7A* | c.700C>T |
| No. | Gene | Variant |  | No. | Gene | Variant |
| 81 | *MYO7A* | c.731G>C |  | 122 | *SLC26A4* | c.1341delG |
| 82 | *MYO7A* | c.1996C>T |  | 123 | *SLC26A4* | c.1343C>A |
| 83 | *MYO7A* | c.2005C>T |  | 124 | *SLC26A4* | c.1343C>T |
| 84 | *OTOF* | c.3624delG |  | 125 | *SLC26A4* | c.1520delT |
| 85 | *PCDH15* | c.1036G>T |  | 126 | *SLC26A4* | c.1540C>A |
| 86 | *PCDH15* | c.1088delT |  | 127 | *SLC26A4* | c.1540C>T |
| 87 | *PJVK* | c.547C>T |  | 128 | *SLC26A4* | c.1541A>G |
| 88 | *SLC26A4* | c.170C>A |  | 129 | *SLC26A4* | c.1547dupC |
| 89 | *SLC26A4* | c.170C>G |  | 130 | *SLC26A4* | c.1554G>A |
| 90 | *SLC26A4* | c.230A>T |  | 131 | *SLC26A4* | c.1555_1556delAA |
| 91 | *SLC26A4* | c.249G>A |  | 132 | *SLC26A4* | c.1586delT |
| 92 | *SLC26A4* | c.259G>T |  | 133 | *SLC26A4* | c.1586T>G |
| 93 | *SLC26A4* | c.281C>T |  | 134 | *SLC26A4* | c.1594A>C |
| 94 | *SLC26A4* | c.349delC |  | 135 | *SLC26A4* | c.1614+1G>A |
| 95 | *SLC26A4* | c.365dupT |  | 136 | *SLC26A4* | c.1615-1G>A |
| 96 | *SLC26A4* | c.367C>T |  | 137 | *SLC26A4* | c.1615-2A>G |
| 97 | *SLC26A4* | c.387delC |  | 138 | *SLC26A4* | c.165-1G>A |
| 98 | *SLC26A4* | c.439A>G |  | 139 | *SLC26A4* | c.1692dupA |
| 99 | *SLC26A4* | c.589G>A |  | 140 | *SLC26A4* | c.1707+5G>A |
| 100 | *SLC26A4* | c.600+2T>A |  | 141 | *SLC26A4* | c.1746delG |
| 101 | *SLC26A4* | c.626G>T |  | 142 | *SLC26A4* | c.1768A>T |
| 102 | *SLC26A4* | c.668T>C |  | 143 | *SLC26A4* | c.1975G>C |
| 103 | *SLC26A4* | c.679G>C |  | 144 | *SLC26A4* | c.1991C>T |
| 104 | *SLC26A4* | c.707T>C |  | 145 | *SLC26A4* | c.1997C>T |
| 105 | *SLC26A4* | c.716T>A |  | 146 | *SLC26A4* | c.2000T>C |
| 106 | *SLC26A4* | c.754T>C |  | 147 | *SLC26A4* | c.2015G>A |
| 107 | *SLC26A4* | c.919-2A>G |  | 148 | *SLC26A4* | c.2027T>A |
| 108 | *SLC26A4* | c.920C>T |  | 149 | *SLC26A4* | c.2089+1G>A |
| 109 | *SLC26A4* | c.1151A>G |  | 150 | *SLC26A4* | c.2162C>T |
| 110 | *SLC26A4* | c.1160C>T |  | 151 | *SLC26A4* | c.2168A>G |
| 111 | *SLC26A4* | c.1173C>A |  | 152 | *SOX10* | c.565G>T |
| 112 | *SLC26A4* | c.1174A>T |  | 153 | *SOX10* | c.621C>G |
| 113 | *SLC26A4* | c.1181_1183delTCT |  | 154 | *TCOF1* | c.386_387delCA |
| 114 | *SLC26A4* | c.1198delT |  | 155 | *TCOF1* | c.422dupA |
| 115 | *SLC26A4* | c.1226G>A |  | 156 | *TCOF1* | c.497_500delATAC |
| 116 | *SLC26A4* | c.1229C>T |  | 157 | *TMC1* | c.100C>T |
| 117 | *SLC26A4* | c.1238delA |  | 158 | *USH1G* | c.113G>A |
| 118 | *SLC26A4* | c.1264-12T>A |  | 159 | *USH1G* | c.84dupC |
| 119 | *SLC26A4* | c.1334T>G |  | 160 | *WFS1* | c.1433G>A |
| 120 | *SLC26A4* | c.1336C>T |  | 161 | *WFS1* | c.1511C>T |
| 121 | *SLC26A4* | c.1341+1G>C |  | 162 | *WHRN* | c.1267C>T |
